# Sit-to-Stand Assistance for Patients with Parkinson’s Disease Using a Lower-Limb Exoskeleton Robot

**DOI:** 10.3390/bioengineering13101226

**Published:** 2026-10-20

**Authors:** Yu-Cheng Wu, Lo-Ping Yu, Si-Huei Lee, Chun-Ta Chen

**Affiliations:** 1 Department of Mechatronic Engineering, National Taiwan Normal University, 162, Section 1, He-Ping East Road, Taipei 106, Taiwan; bihwa00191@gmail.com; 2 Attending Physician of Department of Physical Medicine and Rehabilitation, Taipei Veterans General Hospital, 201, Section 2, Shipai Road, Taipei 106, Taiwan; yuloping@yahoo.com.tw (L.-P.Y.);

**Keywords:** exoskeleton robot, sit-to-stand, Parkinson’s disease, nonlinear disturbance observer (NDO), adaptive fuzzy sliding mode control (AFSMC)

## Abstract

Sit-to-stand (STS) transfer is fundamental to many activities of daily living, but it is difficult for individuals with motor dysfunction or impairment. This study presents an observer-based impedance-control framework for a lower-limb exoskeleton robot to assist STS transfer. To account for external disturbances, including the wearer’s joint torques and model uncertainties, a nonlinear disturbance observer (NDO) is used to estimate the lumped disturbance. An impedance-control strategy based on adaptive fuzzy sliding mode control (AFSMC) is then used to regulate the human exoskeleton system by compensating for these unknown terms. STS trials were conducted in one healthy participant and three participants with Parkinson’s disease (PD), with and without exoskeleton assistance, to evaluate the proposed system. Across ten trials in the healthy participant, O-AFSMC had the lowest mean hip/knee RMSE (2.1247°/2.9375°). In PD participants, assistance reduced CoP and CoM excursion at the 3-s STS condition in each individual; in the H-Y stage-2 participant, CoP excursion decreased from 25.39 to 15.18 cm and CoM excursion from 17.83 to 14.21 cm. These pilot findings support the feasibility of STS assistance with a lower-limb exoskeleton robot.

## 1. Introduction

Sit-to-stand (STS), the transition from a seated to a standing posture, is a fundamental task required for many activities of daily living. STS requires greater lower-limb joint torques than walking because of its high biomechanical demands [1]. Aging, injury, and musculoskeletal or neurological disorders such as stroke and Parkinson’s disease (PD) can impair lower-limb motor function [2,3,4]. Such impairments often compromise STS performance, reducing independent mobility and balance and increasing the risk of falls. Assistive devices can therefore play an important role in supporting this essential movement.

Recent studies of lower-limb exoskeleton robots have demonstrated their potential for powered assistance and rehabilitation [5]. Because a lower-limb exoskeleton is physically coupled to the user, assistance is delivered through human–robot interaction. Depending on the user’s residual muscle strength, STS assistance can generally be divided into two control modes: (1) a user-passive mode, in which the exoskeleton drives the limbs along a preplanned reference trajectory for users who cannot perform the movement independently; and (2) a user-active mode, in which the exoskeleton augments the user’s effort while the user retains partial motor ability [6].

In the user-passive STS mode, intended mainly for users with little or no voluntary mobility, the exoskeleton is responsible for completing the STS transfer without relying on the user’s active contribution. The reference trajectory is typically derived from movement data recorded from healthy individuals and is tracked by the exoskeleton without requiring active coordination from the user’s lower limbs. Robust trajectory tracking control is therefore essential. Sharif et al. [7] proposed a fuzzy controller for lower-limb exoskeletons during sit-to-stand and stand-to-sit movements; however, the controller was evaluated only in simulation using offline and real-time data. Model predictive control (MPC) provides a flexible framework for optimal control. Iqbal [8] applied MPC to STS motion in a multisegment biomechanical model, but the results were limited to simulated STS trajectories.

For practical STS assistance, Shepherd and Rouse [9] developed a powered exoskeleton with series elastic actuators that provides assistive knee extension torque for individuals with stroke. A low-level PID controller combined with a high-level sit-to-stand controller demonstrated the system’s ability to provide assistance, although the study did not report how the assistance level was selected. In [10], a hybrid exoskeleton combining functional electrical stimulation (FES) with a powered exoskeleton was used to assist individuals with mobility impairments during STS. An iterative-learning neural-network control law compensated for structured and unstructured parametric uncertainties to achieve asymptotic tracking of the STS trajectory. However, the use of FES adds complexity to clinical implementation. To accommodate variations in user characteristics and user-generated forces during STS, Mungai and Grizzle [11] developed a quadratic-programming-based computed-torque controller that respects hardware constraints and remains robust to changes in user characteristics. This approach, however, requires constrained optimization to generate feasible dynamic STS motions.

In the user-active STS mode, the exoskeleton must coordinate its assistance with the user’s lower-limb motion. Poor coordination can create antagonistic interaction forces, compromise balance and control, and increase the risk of falling. The exoskeleton must therefore infer the user’s motion intention in real time and provide assistance that complements the user’s residual motor function. Two key challenges are recognizing motion intention and providing safe and stable assistance throughout the STS transfer.

Various sensing modalities have been investigated for recognizing user motion intention, including mechanical sensors [12], surface electromyography (sEMG) [13], and capacitive sensing [14], together with corresponding STS control strategies for human exoskeleton systems. Liu et al. [15] proposed a real-time mode recognition strategy for a bionic knee exoskeleton using one potentiometer and two inertial measurement units to infer the user’s motion intent. Once an STS transition was detected, a three-level controller provided assistive torque to support the movement. Fleischer and Hommel [16] used electromyography (EMG) to measure muscle activity and applied a force amplification scheme to control the exoskeleton. However, EMG signals are weak, difficult to interpret, and sensitive to subject-specific factors [17], which can limit their use in activities of daily living.

More recently, observer-based models and control strategies have been investigated to reduce reliance on additional biosensors while estimating user motion and providing STS assistance to individuals with partial motor ability. Tsukahara et al. [18] inferred motion intention from a preliminary movement observed immediately before the intended action, allowing the exoskeleton to initiate STS assistance. They also developed balance control based on the user’s center of pressure (CoP) and gravity compensation for weight support. However, the threshold-based intention detector was used only to identify STS initiation. Vantilt et al. [19] proposed model-based control to compensate for lower-limb exoskeleton dynamics during STS; however, uncertainty in system parameters may reduce assistance accuracy. Huo et al. [20] developed an impedance-modulation control method that provides power and balance assistance while preserving the user’s residual motor ability. Instead of using sEMG or additional mechanical sensors, human joint torques were estimated with an observer-based model using joint kinematics. Experiments with healthy participants were conducted to validate the STS assistance method.

Recent wearable human–robot interaction research has also investigated compliant pneumatic actuation. For example, the Funabot-Sleeve uses McKibben artificial muscles to generate multiple forearm haptic sensations [21]. Although that device targets upper-limb haptics rather than lower-limb STS rehabilitation, it illustrates the broader role of compliant wearable actuation and physical human–robot interaction.

Despite these advances, most lower-limb exoskeleton systems have been validated primarily in healthy participants. Providing safe STS assistance to patients with neurological or motor impairments remains challenging. Parkinson’s disease (PD) is a degenerative disorder of the central nervous system characterized by symptoms such as tremor, shuffling gait, and bradykinesia. Previous studies have shown that people with PD are slower to initiate and execute movements [22]. STS is particularly challenging because rising from a chair requires coordinated muscle activation and joint motion in the lower limbs and trunk [23].

This study investigates STS assistance for patients with PD using a lower-limb exoskeleton robot. We focus on two factors that can impair STS performance in PD: insufficient balance control and reduced muscle strength. An impedance-control strategy is developed to assist the STS transfer and improve balance while preserving the contribution of users who retain residual voluntary joint torque. Under this control framework, the wearer remains the primary initiator of the STS movement, while the exoskeleton provides appropriate joint torques to reduce the muscular effort required from the user.

## 2. Design and Dynamic Model of the Robotic Exoskeleton

This section briefly describes the proposed hip–knee exoskeleton robot. The exoskeleton must accommodate lower-limb motion during STS, provide the required degrees of freedom for natural movement, and cover the necessary range of motion. The dynamic model of a human wearing the exoskeleton during STS is then formulated.

### 2.1. Hardware and Electronics Overview

**Figure 1:**
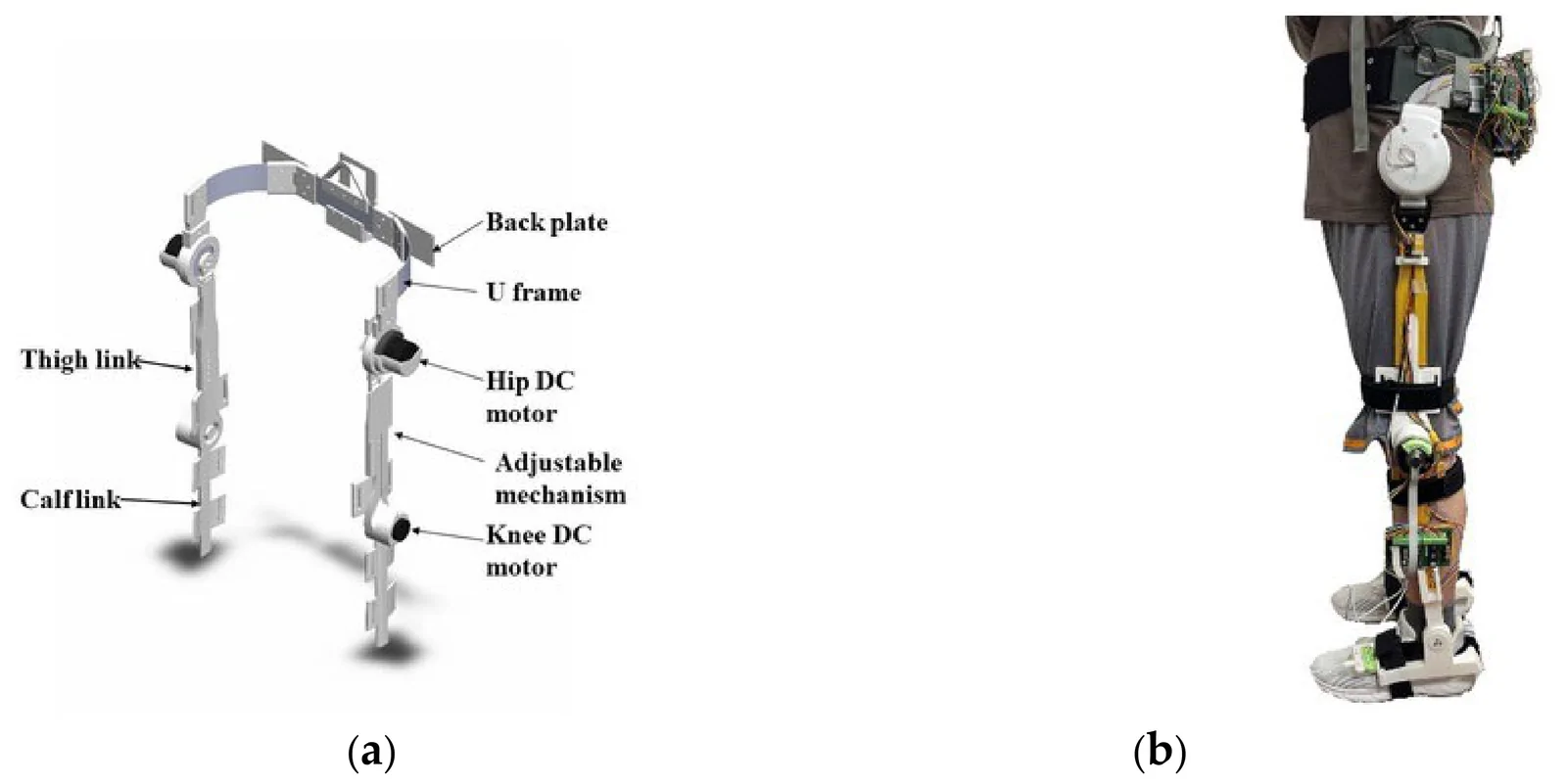
(**a**) Design of the robotic hip–knee exoskeleton. (**b**) Prototype worn by a user.

Each hip joint of the robotic hip–knee exoskeleton provides active flexion/extension driven by a brushless DC motor (MAXON EC 60 flat with nominal current 4.4 A and nominal torque 0.54 Nm) coupled to a 100:1 harmonic reducer, together with passive abduction/adduction for lateral motion. Each knee joint is actuated by a brushless motor (MAXON ECI 52 with nominal current 3.59 A and nominal torque 0.298 Nm) coupled to a 46:1 reducer (IDP Gear 42046), as shown in Figure 1a. Incremental encoders at each active joint measure the joint angles. In addition, a smart insole with 180 distributed force sensors (Lisole Smart Insole) under each foot is used to measure changes in the CoP. A dovetail groove mechanism allows the thigh-link length to be adjusted from 33 to 37 cm to accommodate users of different heights. The links are secured to the lower limbs with multiple straps, and a belt is attached around the waist, as shown in Figure 1b.

**Figure 2:**
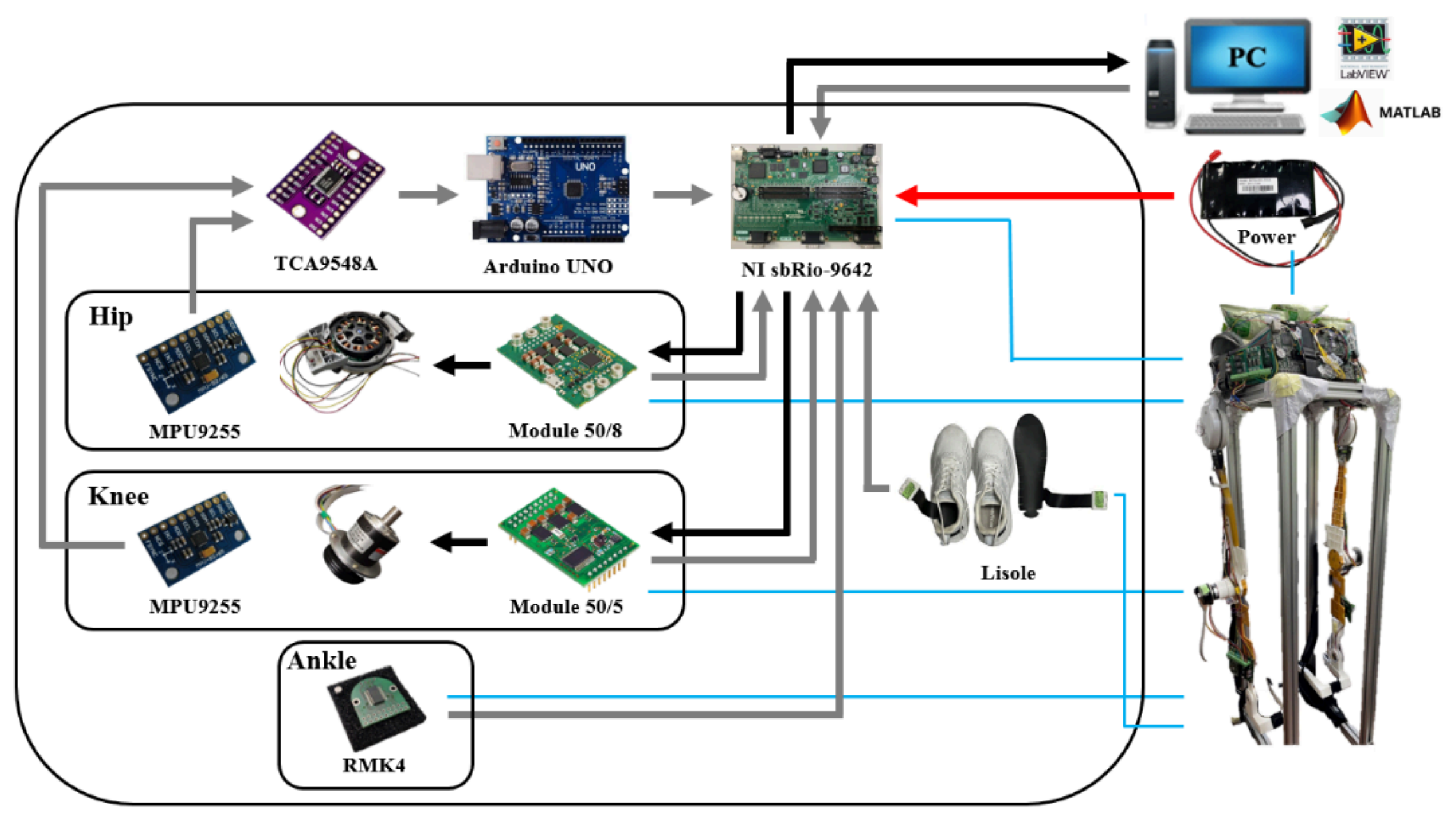
Control circuit and electronic peripherals.

The control circuit and electronic peripherals shown in Figure 2 integrate the actuation units with the main control board. An NI sbRIO-9642, together with reconfigurable I/O (RIO) and FPGA modules, a real-time controller, and an Ethernet expansion chassis, is used to control the robotic exoskeleton. The NI 9234 and NI 9263 modules installed in the cRIO-9072 provide analog-to-digital (A/D) and digital-to-analog (D/A) conversion, respectively. The control algorithms and measurement routines were implemented in LabVIEW.

### 2.2. System Modeling of STS Movement

**Figure 3:**
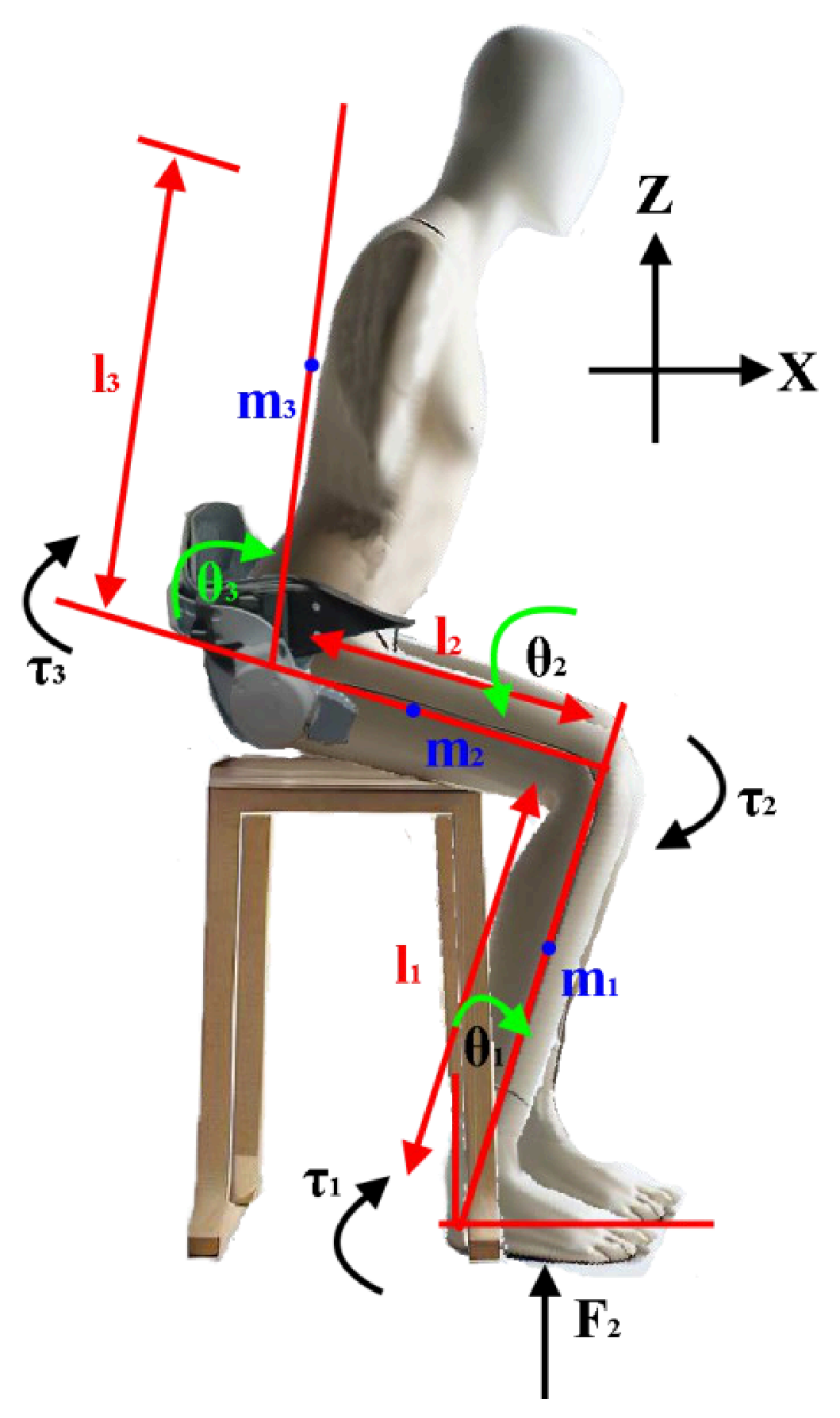
Schematic of the STS movement.

Figure 3 schematically illustrates a person wearing the exoskeleton during STS transfer. The sagittal plane dynamics are modeled as a double inverted pendulum, with tibial dynamics neglected because tibial motion is relatively small during the transition from sitting to standing. The wearer and exoskeleton form a coupled human–machine system; therefore, the physical parameters of the user’s lower limbs and the exoskeleton are combined in the dynamic model. Using the Euler–Lagrange formulation, the dynamics are expressed as

(1) $$(M\left(\theta \right)+\Delta M)\ddot{\theta }+(C\left(\theta ,\dot{\theta }\right)+\Delta C)\dot{\theta }+(G\left(\theta \right)+\Delta H)=u_{e}+u_{h},$$

The joint-angle vector is defined as $\theta ={\left[\begin{array}{ccc} \theta_{1} & \theta_{2} & \theta_{3} \end{array}\right]}^{T}$, where ***θ***_1_, ***θ***_2_, and ***θ***_3_ denote the ankle, knee, and hip joint angles, respectively. ***M*** is the symmetric positive-definite inertia matrix containing the mass and moment-of-inertia terms of the lower limb; ***C***, with $C\dot{\theta }$, is the centripetal–Coriolis matrix containing the joint-velocity-dependent terms; and G represents the gravitational term. **Δ*M***, **Δ*C***, and **Δ*G*** denote structural parameter uncertainties. $u_{e}={\left[\begin{array}{ccc} 0 & \tau_{e,2} & \tau_{e,3} \end{array}\right]}^{T}$ denotes the exoskeleton driving-torque vector, with actuation at the knee and hip joints, and $u_{h}={\left[\begin{array}{ccc} \tau_{h,1} & \tau_{h,2} & \tau_{h,3} \end{array}\right]}^{T}$ denotes the joint-torque vector exerted by the user. Because the reference trajectory begins at the preparation phase (approximately 27% of the STS cycle), whereas seat-off occurs at approximately 34%, chair contact may still be present during the pre-seat-off interval. During the 27–34% transition, the generalized effect of the unmodeled seat reaction is included in the lumped disturbance term ***d*** together with model uncertainty and human-applied torque. Accordingly, Equation (1), which neglects seat reaction, is physically feasible only after the preparation phase.

The parameters of the human exoskeleton system are generally uncertain and different for individuals. The wearer’s joint torques at the ankle, knee, and hip are therefore treated together with model uncertainty as an external disturbance. Equation (1) can be rewritten as

(2) $$M\left(\theta \right)\ddot{\theta }+C\left(\theta ,\dot{\theta }\right)\dot{\theta }+G\left(\theta \right)+d=\tau_{e}$$

where $d=\Delta M\left(\theta \right)\ddot{\theta }+\Delta C\left(\theta ,\dot{\theta }\right)\dot{\theta }+\Delta G\left(\theta \right)-\tau_{h}$ represents the lumped external disturbance. To provide effective STS assistance, an observer-based adaptive fuzzy sliding mode controller is developed for the lower-limb exoskeleton. A nonlinear disturbance observer estimates the external disturbance d, and the AFSMC compensates for this disturbance while tracking the desired STS joint trajectory ***θ_d_*** generated by the impedance model.

## 3. Dynamics and Control of STS Transfer

### 3.1. Observer Design for External Disturbance

To provide effective STS assistance, the nonlinear disturbance observer proposed in [24] is used to estimate the total external disturbance d, including the user’s joint torques ***u_h_*** and system uncertainties. The resulting estimate is then incorporated into the O-AFSMC to compensate for the external disturbance.

The nonlinear disturbance observer is defined as

(3) $$\dot{z}=-L(\theta ,\dot{\theta })z+L(\theta ,\dot{\theta })(\hat{C}\left(\theta ,\dot{\theta }\right)\dot{\theta }+\hat{G}\left(\theta \right)-u_{e}-p(\theta ,\dot{\theta }))$$

(4) $$\hat{d}=z+p(\theta ,\dot{\theta })$$

where $\hat{d}$ denotes the estimated external disturbance vector, ***z*** is an auxiliary variable introduced to avoid direct acceleration measurement, ***L*** is the observer gain matrix, and ***p*** is determined from the modified observer gain matrix such that

(5) $$L\left(\theta ,\dot{\theta }\right)=YM^{-1}(\theta )$$

(6) $$p\left(\theta ,\dot{\theta }\right)=Y\dot{\theta }$$

where ***Y*** is a constant invertible n × n matrix to be determined. To reduce sensitivity to measurement noise while ensuring that the minimum convergence rate of the tracking error is *β*, the LMI formulation and stability analysis yield the following choice of ***Y***:

(7) $$Y=\frac{1}{2}(\zeta +2\beta \sigma )I$$

where ***I*** denotes the identity matrix, *β* is the minimum convergence rate of the human joint tracking error, and *ζ* and *σ* are constants determined by the system dynamics and the maximum joint velocities of the robotic exoskeleton. These constants satisfy

(8) $$\left\|M\left(\theta \right)\right\|\leq \sigma ,\left\|\dot{M}(\theta )\right\|\leq \zeta$$

The nonlinear disturbance observer in Equations (3) and (4) can estimate rapidly varying external disturbances without the conventional assumption that the disturbance derivative is zero.

### 3.2. Observer-Based AFSMC

To accurately track the reference STS motion in the presence of external disturbances, sliding mode control (SMC) with feedforward compensation is adopted. Introducing the reference joint rate ${\dot{\theta }}_{r}={\dot{\theta }}_{d}-\Lambda \tilde{\theta }$, the sliding surface is defined in terms of the joint-rate tracking error. A time-varying sliding surface that specifies the desired closed-loop response is defined as

(9) $$s\left(\theta \right)=\dot{\theta }-{\dot{\theta }}_{r}=\dot{\theta }-({\dot{\theta }}_{d}-\Lambda \tilde{\theta }),$$

where $\tilde{\theta }=\theta -\theta_{d}$ is the joint-angle tracking error, ***θ_d_*** is the desired joint-angle vector, and ***Λ*** is a symmetric positive-definite matrix that determines the desired closed-loop response. The multijoint FSMC with observer-based feed forward compensation is then designed as

(10) $$u\left(t\right)=u_{\mathrm{eq}}\left(t\right)+u_{r}\left(t\right)-\hat{d}\;=M\left(\theta \right){\ddot{\theta }}_{r}+C\left(\theta ,\dot{\theta }\right){\dot{\theta }}_{r}+G\left(\theta \right)+K_{r}\mathrm{FSMC}\left(s,\dot{s}\right)-\hat{d}$$

where the equivalent control ***ueq*** is obtained by setting the derivative of the sliding surface to zero. To reduce the chattering associated with a conventional sign function, the reaching control ur uses an adaptive fuzzy function to smooth the discontinuous control action in the presence of model uncertainties and disturbances. Specifically, the fuzzy reaching term is defined as $K_{r}\mathrm{FSMC}\left(s,\dot{s}\right)={\left[\begin{array}{ccc} k_{r(1)}{\mathrm{FSMC}}_{1}(s_{1},{\dot{s}}_{1}) & \ldots & k_{r(n)}{\mathrm{FSMC}}_{n}(s_{n},{\dot{s}}_{n}) \end{array}\right]}^{T}$, where the FSMC maps the two normalized inputs s(t) and $\dot{s}$(t) to a linguistic output.

**Figure 4:**
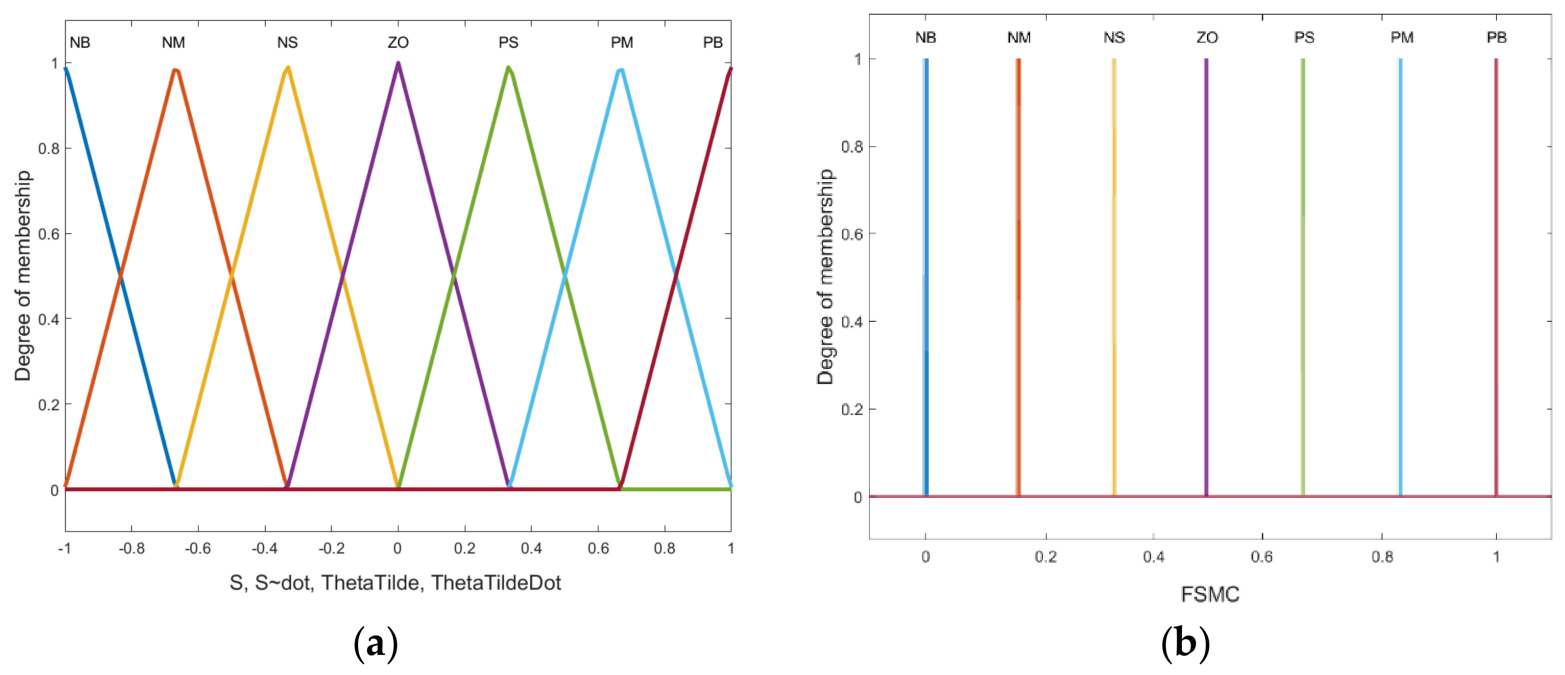
Assigned membership functions of the fuzzy sets for (**a**) the input variables $\left(s_{i},{\dot{s}}_{i}\right)$ and (**b**) the output function ***FSMC_i_***.

As shown in Figure 4, seven fuzzy partitions are defined for the input and output linguistic variables. The fuzzy inference system follows IF-THEN rules of the form *R*^(*i*)^: IF $\tilde{x}$(t) is $A_{1}^{i}$ and $\dot{\tilde{x}}$(t) is $A_{2}^{i}$, THEN *FSMC_i_* is *B^i^*, for the i-th rule, where $A_{1}^{i}$ and $A_{2}^{i}$ are the input fuzzy sets and *B^i^* is the output fuzzy set. The rule base is listed in Table 1. The linguistic labels are NB (Negative Big), NM (Negative Medium), NS (Negative Small), ZO (Zero), PS (Positive Small), PM (Positive Medium), and PB (Positive Big). Singleton fuzzification and centroid defuzzification are used with product inference as in [25]. The fuzzy function is normalized to the interval [−1, 1], such that $\left|{\mathrm{FSMC}}_{i}(s_{i},{\dot{s}}_{i})\right|\leq 1$, and the fuzzy rules are designed to satisfy the condition $s_{i}{\mathrm{FSMC}}_{i}(s_{i},{\dot{s}}_{i}))\leq -\left|s_{i}\right|$.

### 3.3. Stability Analysis

The lower-limb exoskeleton system is controlled by the observer-based FSMC. Consider the Lyapunov candidate *V*(*t*) defined as

(11) $$V\left(t\right)=\frac{1}{2}\left(s^{T}\mathrm{Ms}\right)$$

Differentiating Equation (11) gives

(12) $${\dot{V}}_{1}=\left(s^{T}M\dot{s}\right)+\frac{1}{2}\left(s^{T}\dot{M}s\right)=s^{T}M\left(\ddot{\theta }-{\ddot{\theta }}_{r}\right)+\frac{1}{2}\left(s^{T}\dot{M}s\right)=s^{T}\left(u-C\dot{\theta }-G-d-M{\ddot{\theta }}_{r}\right)+\frac{1}{2}\left(s^{T}\dot{M}s\right)=s^{T}\left(\left[\left(M{\ddot{\theta }}_{r}+C{\dot{\theta }}_{r}+G\right)+K_{r}\mathrm{FSMC}\left(s,\dot{s}\right)\right]-C\left(s+{\dot{\theta }}_{r}\right)-G+\Delta d\left(t\right)-M{\ddot{\theta }}_{r}\right)+\frac{1}{2}\left(s^{T}\dot{M}s\right)\leq \sum_{i=1}^{n}(\left|{\Delta d}_{i}\left(t\right)\right|-k_{r\left(i\right)})\left|s_{i}\right|$$

where ***Δd*** *=* $\hat{d}-d.$ If *k_r_*_(*i*)_ > |Δ*d_i_*(*t*)| is selected such that the reaching condition $\dot{V}\left(t\right)<0$ is always satisfied, the closed-loop system is asymptotically stable.

**Figure 5:**
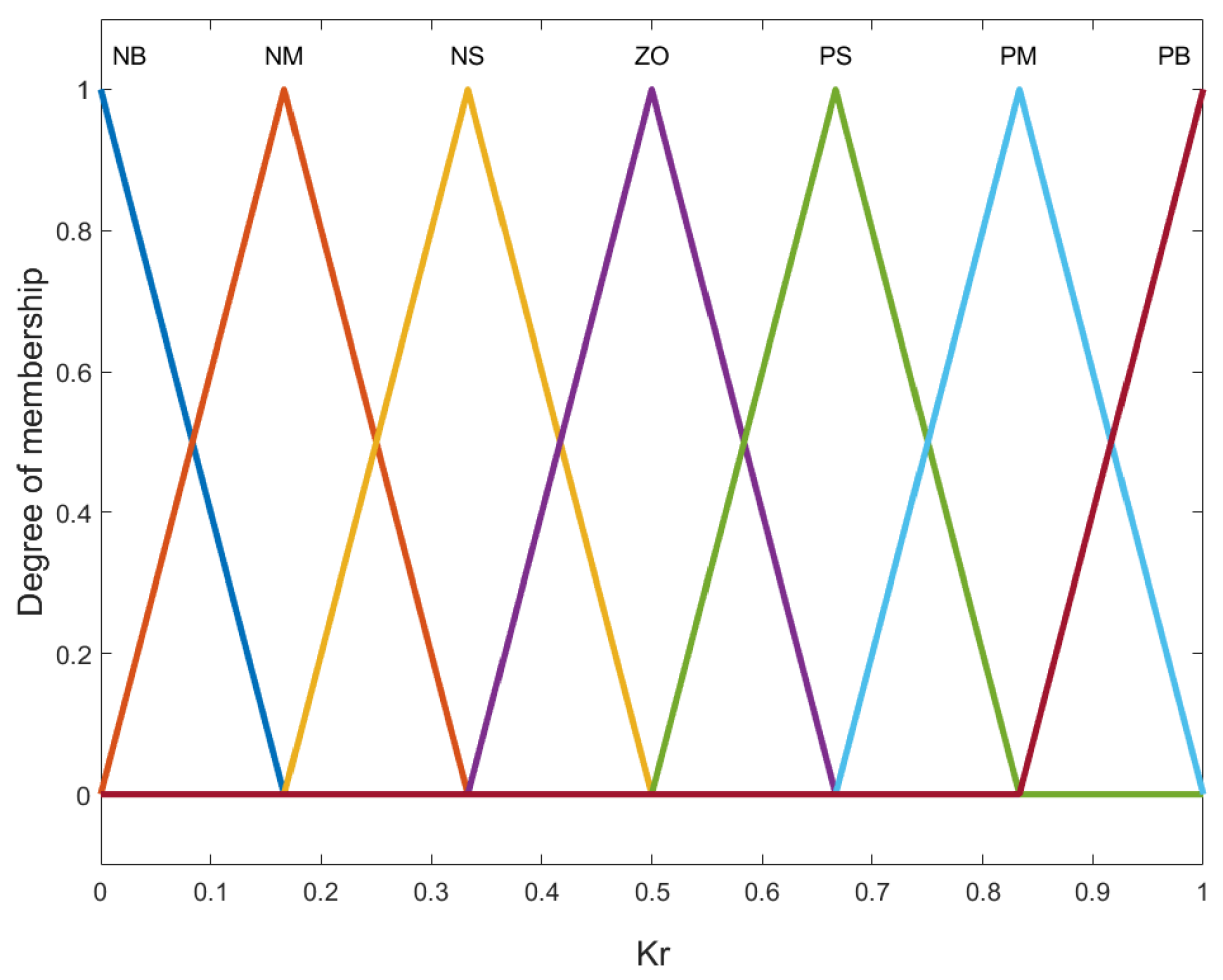
Fuzzy sets for the switching gain ***k_r_***.

The switching fuzzy gains *k_r_*_(*i*)_ in the observer-based FSMC can be adjusted online according to the tracking error to improve STS performance. The gains *k_r_*_(*i*)_ are generated by a fuzzy inference system. Seven fuzzy partitions with triangular membership functions are defined for the two normalized inputs ${\tilde{\theta }}_{i}$ and ${\dot{\tilde{\theta }}}_{i}$ and the output *k_r_*_(*i*)_*,* as shown in Figure 4a and Figure 5. The input–output relationships are specified by the IF-THEN rules in Table 2. Correlation-minimum inference and centroid defuzzification are used. Consequently, *k_r_*_(*i*)_ is adjusted online according to the current joint-angle and joint-rate errors during STS.

### 3.4. Desired Impedance Strategy

Successful STS requires substantial lower-limb joint torques to overcome gravitational and inertial loads. Individuals with neuromusculoskeletal disorders or sensorimotor impairments may be unable to generate sufficient torque. In the proposed STS assistance mode, the wearer remains actively involved in the transfer while the exoskeleton supplies assistive torques that reduce the required effort according to the user’s lower-limb joint torques. An impedance-control approach is therefore adopted to provide assistance while preserving the user’s contribution.

The desired impedance dynamics can be designed according to the wearer’s lower-limb motor ability as

(13) $$M\left(\theta \right){\ddot{\theta }}_{d}+C\left(\theta ,\dot{\theta }\right){\dot{\theta }}_{d}+G\left(\theta \right)=\alpha \hat{d},$$

where ${\dot{\theta }}_{d}$ and ${\ddot{\theta }}_{d}$ are the desired joint velocities and accelerations, respectively, and ***α*** *= diag[α*_1_*, α*_2_*, α*_3_] with ‖***α***‖ ≤ 1 denotes the set of assistance coefficients selected according to the wearer’s lower-limb motor ability. The disturbance estimate provided by the NDO contains the user’s lower-limb joint torques together with system uncertainties. Therefore, Equation (13) is the real desired impedance model to generate the stable reference trajectory. Moreover, the exoskeleton is actuated only at the knee and hip joints; the ankle assistance coefficient is set to *α*_1_ *=* 0.

**Figure 6:**
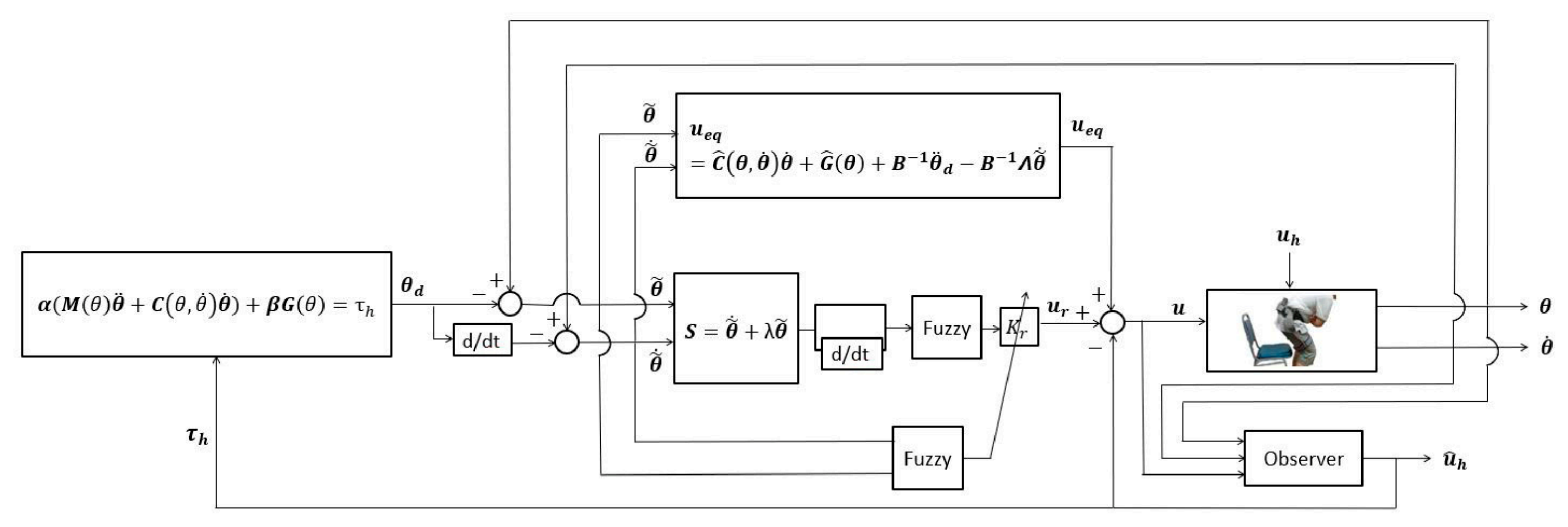
Impedance-control structure with observer-based AFSMC.

The impedance-control structure with observer-based AFSMC for the hip–knee exoskeleton is shown in Figure 6. During user-active STS, the wearer initiates and performs the movement. The NDO estimates the combined effects of the user’s lower-limb joint torques and system uncertainties, and this estimate is fed to the impedance model to generate the reference joint trajectories. The AFSMC then synthesizes the control input u, which drives the DC motors to provide the required assistance.

## 4. Results and Discussion

**Figure 7:**
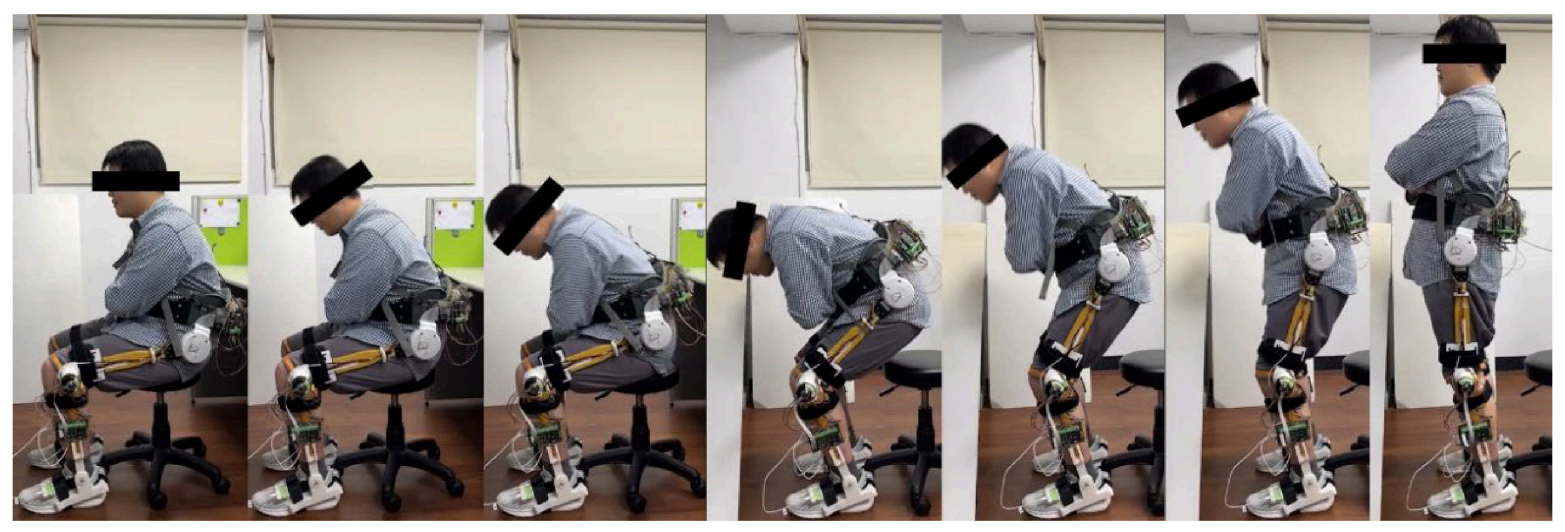
STS experiment with exoskeleton assistance.

This section evaluates the performance of the proposed observer-based AFSMC for STS assistance with the lower-limb exoskeleton. As illustrated in Figure 7, each participant wore an exoskeleton while seated on a chair. The chair height was adjusted for each participant so that the knee flexion angle was approximately 90^°^. Before initiating STS, the participant was asked to flex the trunk forward and then stand with assistance from the exoskeleton. Once full standing was achieved, the joint motor drives were deactivated. For safety, an emergency stop was triggered if the monitored motor current exceeded a preset threshold.

In the implementation, the parameter values for the observer-based controller were selected as the tracking error gain matrix ***Λ*** = ***diag***(0, 0.1, 0.1); the fuzzy-input normalization ranges were set between −1 and 1, and the switching fuzzy gains *k_r_*_(*i*)_ were defined in Table 2 and Figure 5. The parameters related to the NDO were chosen as *ζ* = 80, *β* = 5 and *σ* = 60.

In the hardware, the loop rate of the real-time CPU for the NI controller was set at 3 kHz; all measured data were low-pass filtered at 5 Hz using a fifth-order Butterworth filter. Higher-order derivatives were employed for numerical differentiation. Zero-offset calibration was used for the smart insole and encoder calibration, and the end-to-end latency is 10 ms. The numerical emergency-stop current threshold was set to 4 A.

### 4.1. Assistance Performance During STS Movement

Experiments were conducted with a healthy participant, whose height is 1.70 m, weight 73 kg, and age 30 years old, to compare the STS assistance performance of different controllers. An STS cycle begins at the start point, when the participant is seated in a stable posture. The trunk then flexes forward until the knees are about to extend; this preparation point occurs at approximately 27% of the STS cycle [26]. The exoskeleton reference trajectory was therefore planned from the preparation point onward. Seat-off occurs when the body leaves the chair, and the interaction force between the body and chair becomes zero, at approximately 34% of the cycle. During the subsequent ascent phase, the center of mass (CoM) rises until the hip and knee joints are fully extended; the stabilization point occurs at approximately 73% of the cycle. The STS cycle ends when a stable upright posture is achieved. Throughout the cycle, the hip-, knee-, and ankle-joint angles were recorded using incremental encoders.

Because chair contact remains possible between the preparation point and seat-off, data from the 27–34% interval were interpreted as transition phase data in which seat reaction contributes to the lumped disturbance; post-seat-off interpretation was not affected by this chair-contact term. Moreover, the assistance coefficients were taken α_2_ = α_3_ = 0.2 from the 27–34% interval; afterwards α_2_ = α_3_ = 0.1 because larger assistive torques were needed at a standing start.

**Figure 8:**
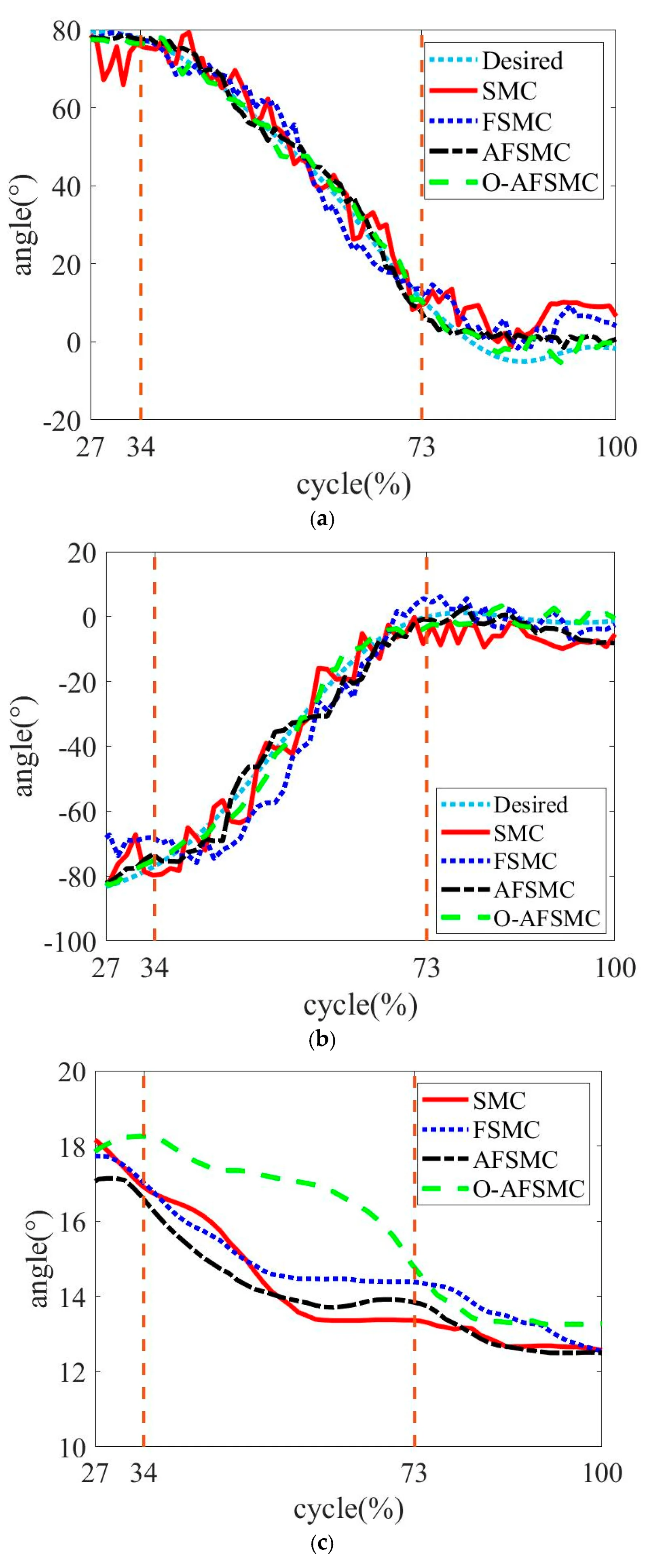
STS trajectories of the (**a**) hip, (**b**) knee, and (**c**) ankle joints under different controllers.

Figure 8a,b show the hip- and knee-joint trajectories, respectively. The planned trajectory was compared with the measured trajectories obtained using four controllers: sliding mode control (SMC), fuzzy sliding mode control (FSMC), adaptive fuzzy sliding mode control (AFSMC), and observer-based adaptive fuzzy sliding mode control (O-AFSMC). SMC converges rapidly to the planned STS trajectory but exhibits pronounced chattering. FSMC reduces much of this chattering, although its tracking performance remains limited. AFSMC further suppresses chattering while maintaining high tracking accuracy. For this healthy participant, O-AFSMC produced the smallest tracking error among the four tested controllers.

Figure 8c shows the ankle-joint trajectories. Across the four control conditions, the participant’s ankle angle varied by approximately 4 degrees. The O-AFSMC trajectory differs from those obtained with the other controllers because the observer reduces chattering at the actuated hip and knee joints, which in turn affects the resulting ankle motion.

Table 3 reports the mean root mean square error (RMSE) over ten trials for the four controllers, where RMSE = $\sqrt{\frac{\sum_{j=1}^{n}{\left(\theta_{j}-\theta_{\mathrm{jd}}\right)}^{2}}{n}}$, *θ_j_* is the measured joint angle, and *θ_jd_* is the planned joint angle. For this participant, O-AFSMC yielded the lowest mean hip- and knee-joint RMSE values among the four controllers (2.1247° and 2.9375°, respectively). Because all repeated trials were obtained from one individual, this descriptive comparison does not establish population-level superiority.

**Figure 9:**
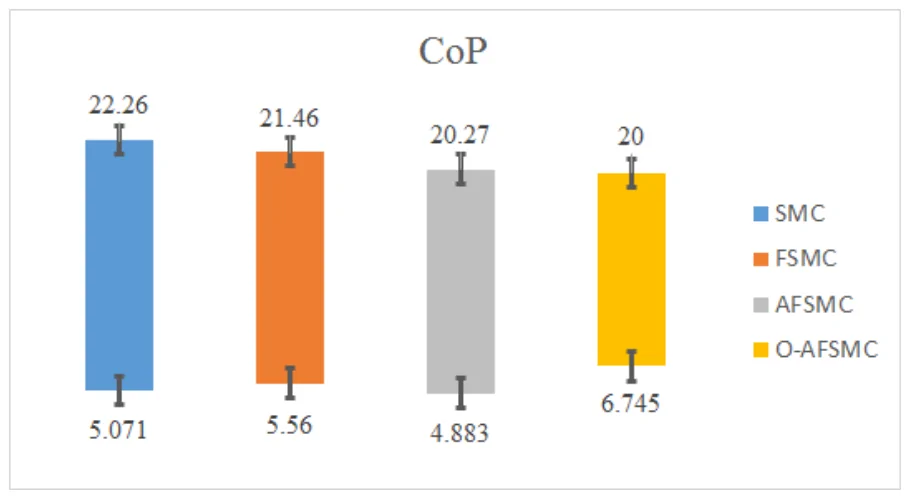
CoP variation under different controllers.

Figure 9 shows the CoP variation obtained with the different controllers during STS. The posterior-most point of the participant’s foot is defined as the origin, and the anterior-most point is 28 cm from the origin. The measured CoP ranges for SMC, FSMC, and AFSMC are 5.071–22.26 cm, 5.56–21.46 cm, and 4.883–20.27 cm, respectively. The O-AFSMC range is 6.745–20 cm. For this participant, O-AFSMC produced the smallest CoP excursion among the four controllers. This smaller excursion is treated as a preliminary balance-related indicator.

**Figure 10:**
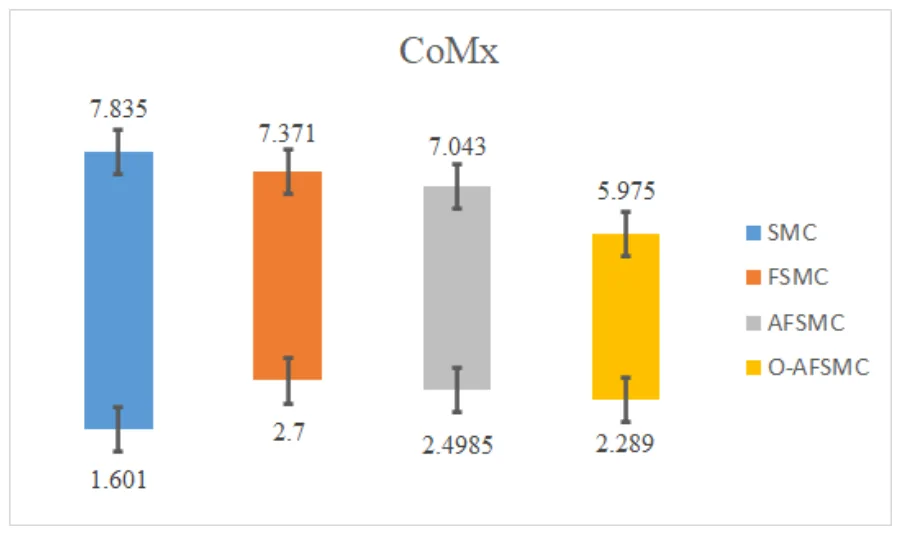
CoM_x variation under different controllers.

Figure 10 shows the horizontal center-of-mass position (CoM_x) of the human exoskeleton system. The posterior-most point of the participant’s foot is defined as the origin (0 cm), with the anterior direction positive and the posterior direction negative. The measured CoM_x ranges for SMC, FSMC, AFSMC, and O-AFSMC are 1.601–7.835 cm, 2.7–7.371 cm, 2.4985–7.043 cm, and 2.289–5.975 cm, respectively. For this participant, O-AFSMC produced the smallest CoM_x excursion among the four controllers. This is consistent with reduced horizontal sway.

### 4.2. STS Assistance for Patients with PD

**Figure 11:**
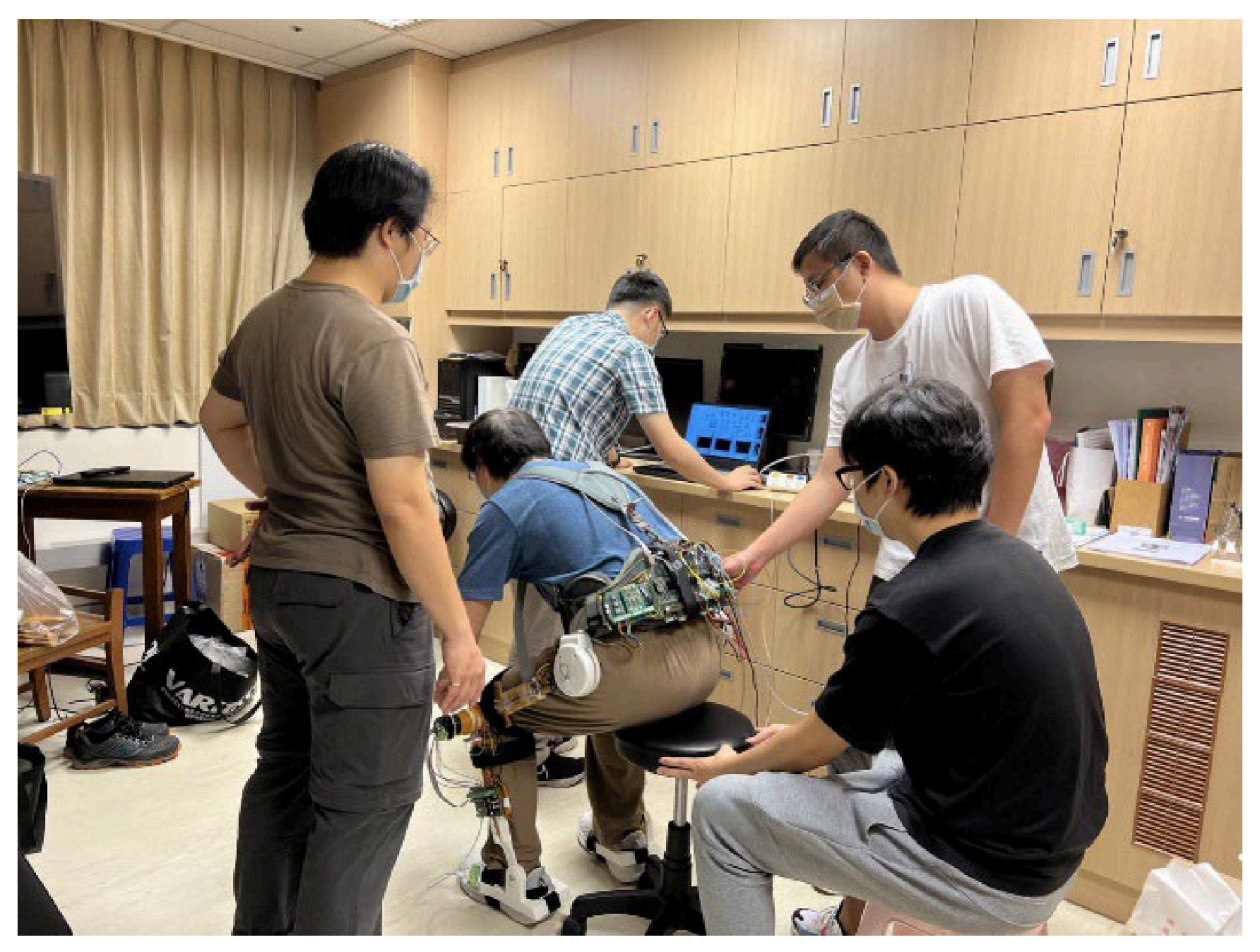
Clinical STS trials with the exoskeleton robot in participants with PD.

The study involving patients with PD and the proposed lower-limb exoskeleton was approved by the TMU-Joint Institutional Review Board, and informed consent was obtained from all participants and their family members. The experiments were conducted in the Technology Medical Rehabilitation Room of the Integrated Rehabilitation Center at Taipei Veterans General Hospital. Three participants with Hoehn and Yahr (H-Y) stages 1, 1.5, and 2 [27] were recruited, as shown in Figure 11. During the clinical trials, the participants retained primary control of the STS movement and received assistance through the impedance assistance mode using O-AFSMC. The recorded outcomes included joint angles and CoM and CoP positions. The clinical pilot included one participant at each H-Y stage (1, 1.5, and 2); therefore, between-stage patterns are presented only as individual observations and are not yet used for statistical inference across H-Y stages. The participants’ characteristics are presented in Table 4. The assistance coefficients for the PD pilots on STS assistance were taken α_2_ = α_3_ = 0.3 from the 27–34% interval, and afterwards α_2_ = α_3_ = 0.2.

**Figure 12:**
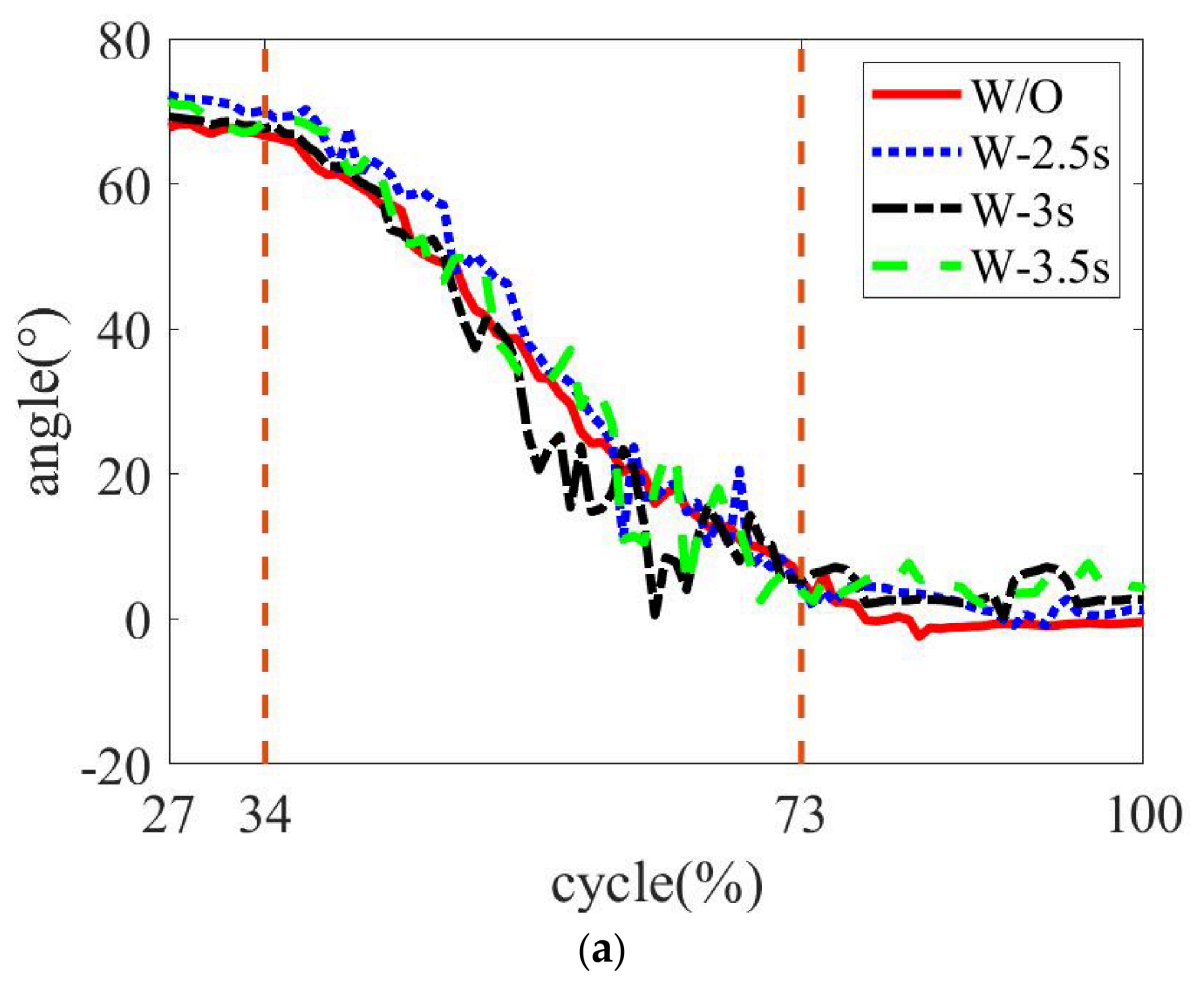

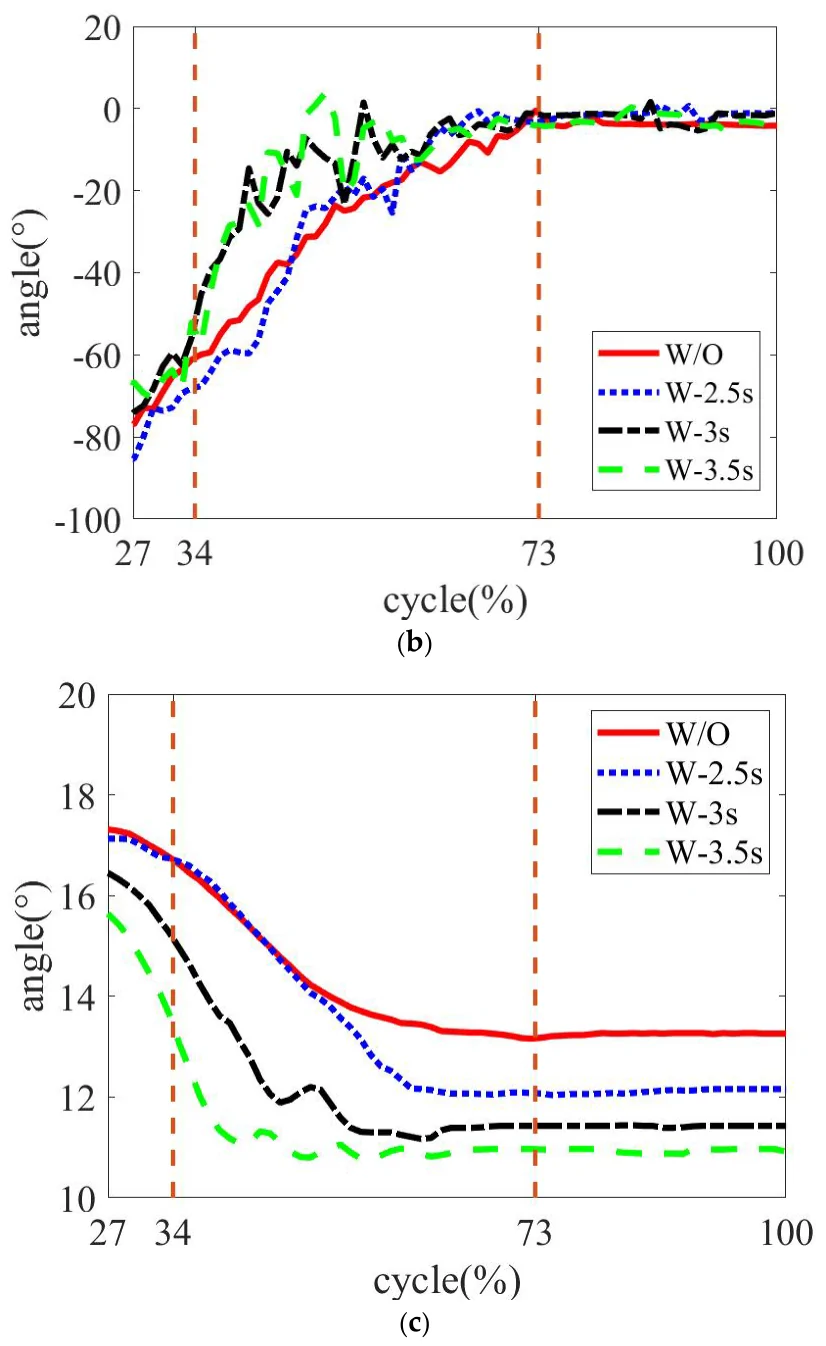
STS trajectories of the (**a**) hip, (**b**) knee, and (**c**) ankle joints at three STS durations for a participant with H-Y stage 1.

Three target STS durations, 2.5, 3.0, and 3.5 s, were evaluated with exoskeleton assistance. An STS duration of approximately 3 s is commonly observed in healthy individuals. Before formal data collection, the participants practiced the task several times to become familiar with the target durations. Figure 12a–c show the hip-, knee-, and ankle-joint trajectories for the participant with H-Y stage 1. With exoskeleton assistance, the trajectories exhibit noticeable oscillations caused by antagonistic interaction between the participant and the exoskeleton, together with the participant’s inherent postural instability and tremor. Table 5 lists the RMSE values relative to the impedance model reference trajectories for the three STS durations. The shorter the STS duration, and therefore the faster the movement, the larger the tracking error. This reflects the greater difficulty of coordinating human and exoskeleton motion during faster STS transfers.

**Figure 13:**
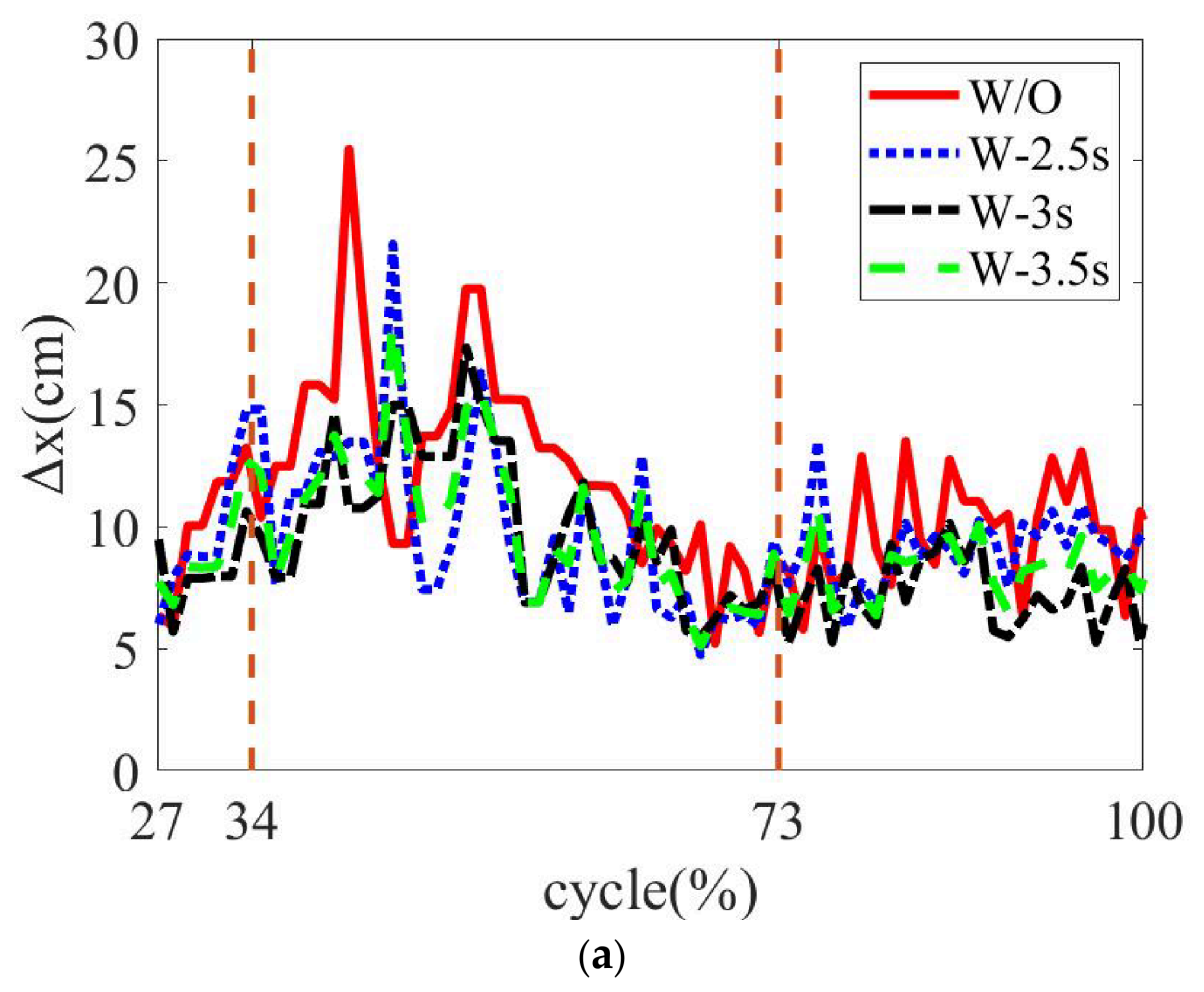

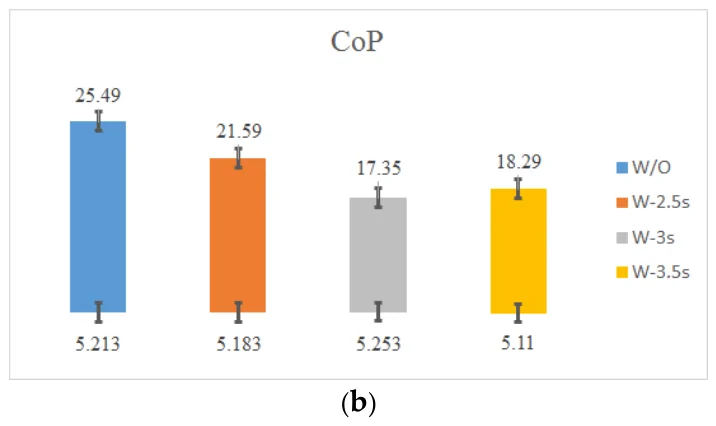
CoP variation at different STS durations: (**a**) trajectories over the movement cycle, and (**b**) bar chart.

Figure 13a shows the CoP variation during STS. During the initial forward trunk flexion, body weight is supported mainly by the chair. As standing proceeds, the trunk extends, and body weight is gradually transferred to the lower limbs. After seat-off, the lower limbs support the full body weight. In the early phase of STS, the CoP shifts anteriorly. Without assistance, the CoP moves farther forward, which may compromise stability if it approaches or exceeds the support boundary. In addition, PD-related postural instability [28] reduces the ability to maintain standing balance, resulting in larger CoP fluctuations near the end of the movement under the unassisted condition. Figure 12b summarizes the CoP ranges for the different STS durations. In the three tested individuals, CoP excursion was smaller with assistance than without assistance. This descriptive difference suggests reduced anterior–posterior CoP motion. A longer, slower STS movement was also associated with smaller CoP excursion in the reported individual data.

**Figure 14:**
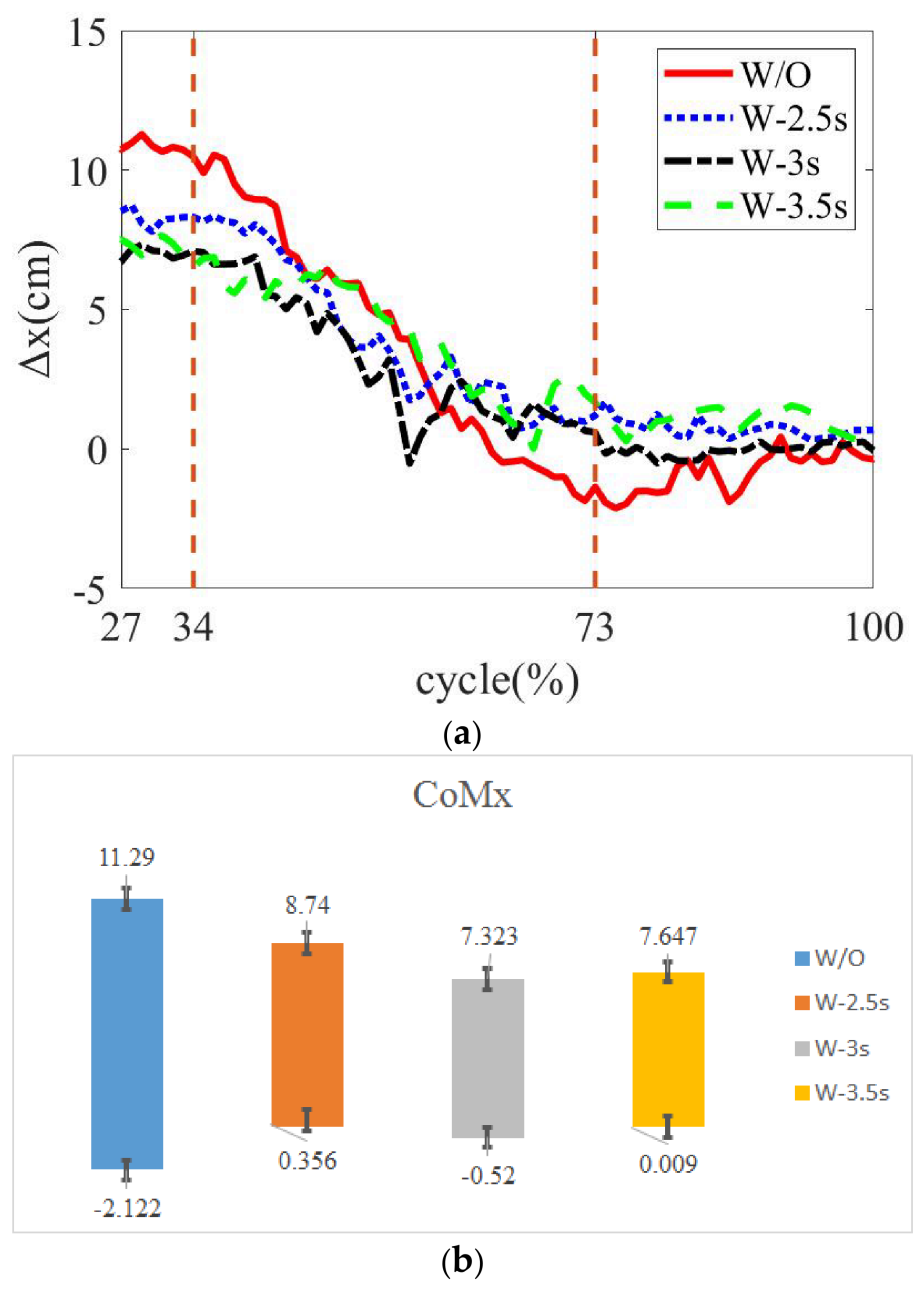
CoM variation at different STS durations: (**a**) trajectories over the movement cycle, and (**b**) bar chart.

Figure 14a shows the horizontal CoM displacement along the *x*-axis for the different STS durations. The maximum anterior displacement occurs near the initiation of rising because forward trunk flexion shifts the CoM toward the front of the base of support. As standing continues, the CoM moves toward a stable upright position over the feet. From the initial standing phase (73% of the cycle) to the final stabilization phase (100%), the unassisted CoM fluctuates more than the CoM measured with exoskeleton assistance. The ability to transfer the CoM from sitting to a stable upright posture is important for independent functional activity, and modifying the CoM trajectory during STS redistributes mechanical demand across the lower-extremity joints [29]. As shown in Figure 13b, exoskeleton assistance reduced the magnitude of CoM displacement in the tested individuals. CoM excursion is interpreted as a descriptive postural control indicator and not as a standalone measure of dynamic balance.

**Figure 15:**
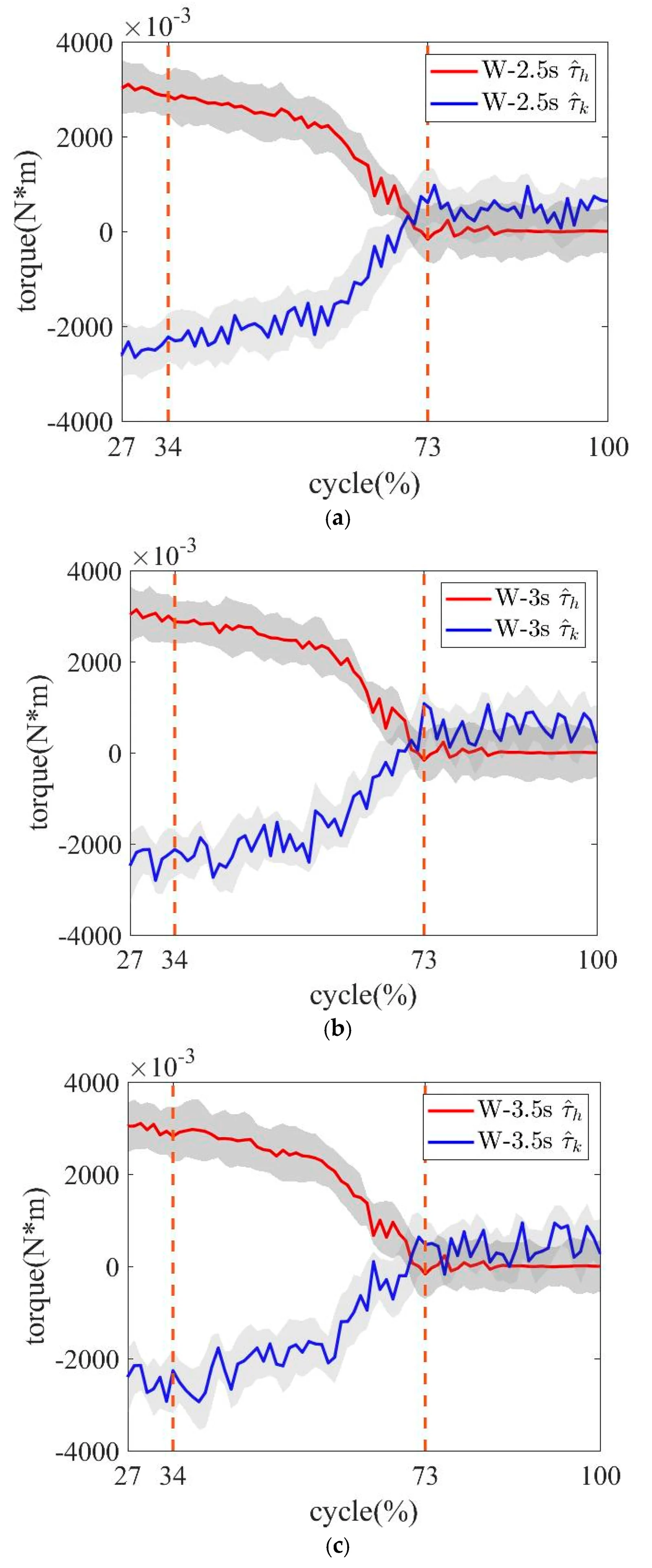
Assistive hip- and knee-joint torques at different STS durations with (**a**) 2.5 secs, (**b**) 3.0 secs, and (**c**) 3.5 secs.

Figure 15 shows the assistive hip and knee torques for the three STS durations. Because the exoskeleton follows the participant’s movement during STS, the torque profiles broadly correspond to the hip- and knee-joint trajectories, *θ_h_* and *θ_k_*. The largest assistive torques are provided during the initial phase of STS and then decrease toward zero as the participant reaches full standing with the lower limbs extended. Larger assistive torques are required for the slower STS condition. During the stabilization phase, balance is maintained with small fluctuations in the assistive hip and knee torques.

### 4.3. Comparison of STS Performance Across H-Y Stages

In this subsection, user-active STS trials were conducted in three individual participants with H-Y stages 1, 1.5, and 2, respectively, using a target STS duration of 3 s. The results are summarized in Table 6. Descriptively, hip- and knee-joint RMSE values were lowest in the H-Y stage 1 participant and higher in the stage 1.5 and 2 participants. Without assistance, CoP excursion was 20.27, 22.83, and 25.39 cm for the H-Y stage 1, 1.5, and 2 participants, respectively; with assistance, the corresponding values were 12.01, 13.21, and 15.18 cm. CoM excursion showed a similar individual-level pattern.

### 4.4. Comparison with Previous Studies

Previous STS assistance studies have used different hardware and control strategies. Shepherd and Rouse [9] demonstrated torque-controllable knee extension assistance for individuals with stroke, in which assistance level needed to be selected. In contrast, Huo et al. [20] used observer-based impedance modulation and evaluated STS assistance in healthy participants. The present study differs by combining NDO-based lumped disturbance estimation with impedance-based O-AFSMC and by including a small PD clinical pilot. STS assistance was based on the evaluated lumped disturbance from the NDO to compensate for the required STS torques without a selected assistance level.

## 5. Conclusions

This study implemented an impedance-control strategy based on AFSMC for a lower-limb exoskeleton robot to assist STS transfer in users who retain partial motor function but cannot generate sufficient effort for reliable STS. The desired impedance compensation is adjusted using the NDO-derived lumped disturbance. The proposed observer-based AFSMC was first evaluated with the lower-limb exoskeleton in the user-passive mode. In the single healthy participant, O-AFSMC descriptively produced the lowest mean hip- and knee-joint tracking errors among SMC, FSMC, AFSMC, and O-AFSMC and also showed the smallest CoP and CoM excursions.

In the user-active trials with participants with PD, the effect of STS duration on balance was evaluated using the impedance-control strategy. In the three enrolled participants, a slower target STS duration was associated with smaller tracking errors in the reported individual data, and exoskeleton assistance reduced CoP and CoM excursion in each participant.

Although these within-participant results support technical feasibility, they do not establish population-level superiority. In addition, although a comparison of STS performance across H-Y stages with the lower-limb exoskeleton robot was made, only one participant represented each H-Y stage; larger clinical tests are required to draw more stage-dependent conclusions.

Given the pilot sample used in this study, the technical feasibility from the individual-level observations is demonstrated, and population-level efficacy or disease-stage effects will be further inferred. Therefore, the population-level controller superiority or H-Y-stage effects will be established. Future work will include larger, more detailed clinical trials and incorporate additional balance models to extend assistance to patients with more advanced PD and to support a broader range of activities of daily living (ADL). Hardware modifications will also focus on improving structural robustness and ergonomic fit to reduce mechanical interference between the user and the exoskeleton. In addition, future validation will require a larger PD cohort, independent validation of the NDO estimate against direct or independently derived human-joint-torque measures, reproducible selection and sensitivity analysis of the assistance coefficients, randomized or counterbalanced trial order with appropriate statistical analysis, and expanded biomechanical balance metrics beyond CoP/CoM excursion.

## Figures and Tables

**Table 1:** Fuzzy rules for FSMC.

FSMC	*s*						
$\dot{s}$	NB	NM	NS	ZE	PS	PM	PB
NB	PB	PB	PB	PB	PM	PS	ZE
PM	PB	PB	PB	PM	PS	ZE	NS
NS	PB	PB	PM	ZE	ZE	NS	NM
ZO	PB	PM	PS	ZE	NS	NM	NB
PS	PM	PS	ZE	ZE	NM	NB	NB
PM	PS	ZE	NS	NM	NB	NB	NB
PB	ZE	NS	NM	NB	NB	NB	NB

**Table 2:** Fuzzy rules for *k_r_*_(*i*)_.

*k_r_*_(*i*)_	${\tilde{\theta }}_{i}$						
${\dot{\tilde{\theta }}}_{i}$	NB	NM	NS	ZO	PS	PM	PB
NB	PB	PB	PB	PB	PM	PS	ZO
PM	PB	PB	PB	PM	PS	ZO	NS
NS	PB	PB	PM	ZO	ZO	NS	NM
ZO	PB	PM	PS	ZO	NS	NM	NB
PS	PM	PS	ZO	ZO	NM	NB	NB
PM	PS	ZO	NS	NM	NB	NB	NB
PB	ZO	NS	NM	NB	NB	NB	NB

**Table 3:** RMSE for different controllers.

	Hip Joint	Knee Joint
SMC	6.5389 (°)	6.9841 (°)
FSMC	5.0986 (°)	5.4463 (°)
AFSMC	3.0452 (°)	3.4552 (°)
O-AFSMC	2.1247 (°)	2.9375 (°)

**Table 4:** PD participants’ characteristics on STS transfer.

	Age	Gender	Y-H Stage	PD Duration (Years)	Height (m)	Weight (kg)	Medication State	Baseline STS Ability
A	71	F	1	5	1.62	52	Yes	normal
B	72	M	1.5	2	1.67	63	Yes	normal
C	64	M	2	3	1.72	72	Yes	normal

**Table 5:** RMSE for different STS durations.

	Hip Joint	Knee Joint
W-2.5 s	1.7846°	1.4639°
W-3 s	1.545°	1.2715°
W-3.5 s	1.2642°	1.1697°

**Table 6:** Comparison of STS performance across H-Y Stages.

	H-Y Staging	1		1.5		2	
		W/O	W	W/O	W	W/O	W
RMSE-Hip (°)		—	1.54	—	1.78	—	2.01
RMSE-Knee (°)		—	1.27	—	1.50	—	1.82
ΔCOP (cm)		20.27	12.01	22.83	13.21	25.39	15.18
ΔCOM (cm)		13.41	12.33	15.16	12.58	17.83	14.21

## Data Availability

The data that support the findings of this research are available from the corresponding author, [C.T. Chen], upon reasonable request.
